# The Approaching Dental Congress in Paris

**Published:** 1889-06

**Authors:** 

**Affiliations:** 24 Rue de la Chaussèe, d’Antin, Paris, France


### Editorial.

### THE APPROACHING DENTAL CONGRESS IN PARIS.

In the "Foreign Correspondence" department in this issue, appears a long letter from our late lamented confrerѐ and correspondent, Dr. E. Brasseur, relating to the International Dental Congress, to be held in Paris in September, and to which all American dentists, who desire the advancement of their chosen profession, are cordially invited.

We have refrained from saying anything in regard to the Congress, preferring to allow our foreign correspondents the privilege of setting forth in our columns the merits of their claims to American support, and this, we are happy to say, they have ably done. We are heartily in favor of supporting the coming Dental Congress, and feel safe in assuring our readers of abundant hospitality and a profitable time, should they desire to attend its sessions this fall.

We offer our support to the Congress on the basis of its object, as explained by its foremost promoters, Drs. Brasseur, Dubois, and Kuhn, who disclaim any intention on their part to supplant in any way the dental section in the Tenth International Medical Congress to be held in Berlin. This is unequivocally stated in their letters published in this journal. We feel called upon to make this statement, because of an editorial which appeared in the *Dental Review*, of Chicago, for May, in which it is said that "an attempt will be made to secure the Second International Dental Congress for the Auditorium Building, Chicago, September 1st, 1892." If this be done it will cripple, if not entirely destroy, American interest in the dental section of the next medical congress in Berlin. Should the *Review* persists in its efforts to call a return congress here in 1892, it will work an irreparable injury to the present congress. The notion of continuing the French congress, as a separate Dental Congress, is entirely foreign to the intentions of its promoters. They consider it simply at a part of a number of bodies that have been convoked at Paris, because of the excellent opportunity afforded for doing so by reason of the large number of professional men that will, in all probability, attend the exposition this year.

Dr. Brasseur states very plainly how it came to be called International. There is no mistaking the tenor of his letter. He most disqualifiedly denied any intention on his part, or that of his confrerés, in any way to interfere with or supplant the dental section of the next International Medical Congress. Neither is Dr. Dubois any less frank in his denial; for he says: "In assembling this congress the French dentists do not wish to interfere in any way with the dental section of the International Medical Congress to be held in Berlin." Dr. Kuhn also says in his letter, published in the April number of the *Journal:* "I can boldly affirm that this idea (of interfering with the Berlin congress) has never entered the mind of any one among us in France." These statements are so frank and open, that no one can doubt the candor of those who make them. And if the matter is allowed to drop here a full contingent of American dentists will be in attendance at the congress; but we predict, that if the *Review* continues to push its pet plan of calling a return congress, there will spring up a strong feeling of opposition, which will show itself during the remainder of the summer and fall. American dentists have struggled too long to obtain medical recognition, to submit now to be thwarted, in order to please the ideas of a very small faction in America.

We pledge our hearty support to the French Congress, as explained by its promoters, Drs. Dubois, Kuhn and Brasseur. This we have done in the past, in allowing the free use of our columns for a full presentation of the subject, and will still continue so to do. On the other hand, we shall strenuously oppose the calling of an independent dental congress in America, and shall use our utmost endeavors to prevent it. The calling of a congress, however, is one thing, while making of it a success, is quite another. We think that the *Review* has perhaps been slightly precipitate in designating the new Aditorium Building as the meeting place for the return congress, as we are credibly informed that its seating capac- is only 5,000, which may not be great enough to accommodate all who may desire to attend. In the mean time, active preparations are going on for the coming congress, and all who wish to present communications should send them as soon as possible to the Secretary.

M. Pourchet,
24 Rue de la Chaussèe, d'Antin, Paris, France.

